# Late preterm births: a retrospective analysis of the morbidity risk stratified for gestational age

**DOI:** 10.1186/2193-1801-3-114

**Published:** 2014-02-28

**Authors:** Sonia Marrocchella, Veronica Sestilli, Ugo Indraccolo, Filomena de Rosario, Lara Castellana, Anna Lucia Mastricci, Anna Calo’, Rosario Magaldi, Antongiulio Del Bianco, Pantaleo Greco, Maria Matteo

**Affiliations:** Department of Medical and Surgical Sciences, Institute of Obstetrics and Gynecology, University of Foggia, Viale Pinto, 71100 Foggia, Italy; Operative Unit of Gynecology and Obstetrics of Civitanova Marche, Area 249 Vasta 3, Marche, Italy; Department of Biomedical Sciences, University of Foggia, Foggia, Italy; Operative Unit of Neonatology and Neonatal Intensive Care Unit – Ospedali Riuniti di Foggia, Foggia, Italy

## Abstract

**Purpose:**

Late-preterm births are considered functionally mature but, several line of evidences suggest that, compared with term neonates, they have a higher risk of complications. The aim of this study was to compare the incidence of maior clinical complications of late preterm infants born in our division, compared to those born at term.

**Methods:**

We retrospectively analysed late preterm deliveries occurred in a twenty-months period. Late preterms were divided in 3 sub-groups according to gestational age at delivery: 34 0/6 , 35 0/6 , 36 0/6 weeks of gestation. The incidence of maior clinical complications was evaluated. Statistical analysis was performed by using the Z- test.

**Results:**

Among term deliveries 17.24% were admitted to the neonatal intensive care unit and 69.01% presented one major adverse outcome: 25.35% jaundice, 25.35% hypoglycemia , 11.26% RDS , 4.22% intraventricular hemorrhage (IVH), 4,22% anemia. The incidence of IVH was significantly higher only at 340/6 weeks of gestation compared to term infants. The incidence of anemia and RDS was significantly higher at 34 0/6 and 35 0/6 weeks of gestation, but it was not significantly different at 36 weeks of gestation, compared to full-term infants. Finally, the incidence of hypoglycemia and jaundice results significantly higher in all the 3 sub groups of late preterms, compared to full term infants.

**Conclusions:**

Results demostrated an increased risk of morbidity in the late preterm period. Results also showed that the gestational age at delivery of late preterms can influence the risk of adverse neonatal outcomes.

## Introduction

Preterm labour is usually defined as regular, painful, synchronous uterine contractions accompanied by cervical change before 37 completed weeks of gestation (Gotsch et al. 2009). The pathogenesis of preterm birth is still under debate; in fact preterm labour might be the result of the early idiopathic activation of the normal labour process or the consequence of pathological insults (Papatsonis et al. 2009). In industrialized countries, the incidence of the preterm delivery is generally 5–9% (Goldenberg et al. 2008) and it represents one of the serious problems in perinatal medicine representing the most important cause of perinatal morbidity and mortality after congenital anomalies (Papatsonis et al. 2009). The preterm birth rate has increased by 33% in the last 25 years, almost entirely due to the rise in late preterm births defined as births between 34^0/7^ and 36^6/7^ weeks of gestation (Committee on Obstetric Practice 2008)*.* Late-preterm births are about 74% of all preterm births and about 8% of total births (Davidoff et al. 2006). These infants are often considered both physiologically and metabolically “functionally mature” because they are of similar size and weight at birth as infants born at term, indeed they seem to have less severe neonatal complications and less long-term neurological sequelae when compared to infants born before 34 weeks of gestation (Abe et al. 2010). However recently attention has been given to uncovering the often subtle morbidity risk associated with late preterm delivery (Shapiro-Mendoza & Lackritz 2012)*.* In fact, although little published information exists regarding morbidities of late preterm infants, the available evidence suggest that, when compared with term neonates, late-preterm are at higher risk of developing medical complications such as temperature instability, hypoglycemia, respiratory distress (RDS), apnea, anemia, jaundice, feeding difficulties, apnea, intraventricular hemorrhage (IVH), seizures, necrotizing enterocolitis (Bastek et al. 2008; Loftin et al. 2010). Moreover late preterm have higher rates of hospital readmission in the first months of life (Committee on Obstetric Practice 2008) and a higher mortality rate during infancy (Consortium on Safe Labor et al. 2010) suggesting that the neonatal risk when delivery occurs between 34 and 37 weeks of gestation is not negligible. On these basis the aim of this study was to compare the incidence of major clinical complications of late preterm infants born, in our division with those born at term. Moreover we also evaluated the incidence of neonatal complications in three different subgroups, stratified for gestational age, in order to estimate the contribution of each week of gestation at delivery to neonatal morbidity.

## Materials and methods

We retrospectively analyzed all late preterm deliveries (34 ^0/6^ to 36 ^0/6^ weeks of gestation) occurred in the Department of University Obstetrics and Gynecology of Foggia, Italy, in twenty-month period. The study was conducted in accordance with the guidelines in the Declaration of Helsinki and was approved by the committee of the Department of Medical and Surgical Sciences of the University of Foggia. Written informed consent was obtained from the patient’s guardian/parent/next of kin for the use of clinical data in order to search and for the publication of this report and any accompanying images.

The study group was compared with a control group consisting of full-term deliveries. Gestational age was determined by the first day of mother’s last normal menstrual period (LMP) with confirmatory ultrasonografy (US). When last menstrual period was unknown, dating was assigned by earliest US. The late preterm group was divided in 3 sub-groups: Group A: 34 ^0/6^, Group B: 35 ^0/6^, Group C: 36 ^0/6^. None of patients enrolled had taken corticosteroid treatment before delivery. Abnormal pregnancies such as gestational diabetes, pregnancy related hypertension, placenta praevia and other medical and obstetrical disorders were excluded from the study. Admission criteria to the Neonatal Intensive Care Unit (NICU) include any of the following: hypoglycemia, jaundice, intraventricular hemorrhage, respiratory distress requiring respiratory support for longer than 24 hours, need for total parenteral nutrition, suspected sepsis, or significant hematologic abnormality (i.e., anemia, polycythemia, or thrombocytopenia), requirement for close observation as assessed by a neonatologist. Firstly, it was assessed if late preterm infants have a higher admission rate in NICU. Then, among late preterm sub-groups and full-term infants, it was assessed the rate of the major adverse outcomes as follows: hypoglycaemia: blood glucose less than 50 mg/dl (Bastek et al. 2008); intraventricular haemorrhage grades I-IV based on the extent of hemorrhage (Papile et al. 1978) , jaundice: hyperbilirubinemia requiring phototherapy (Petrova et al. 2006), respiratory distress syndrome/hyaline membrane disease (RDS) was typically defined as respiratory symptoms (eg, grunting, flaring, tachypnea, retractions), supplemental oxygen requirement, and NICU admission for further respiratory support, with the diagnosis verified by chest radiograph findings of reticulogranular patterns and air bronchograms (The Consortium on Safe Labor 2010), anemia: hemogolobin levels requiring transfusion.

### Statistical analysis

Statistical analysis was performed by using the Z- test. An alpha value of 0.05 was used for assessing statistical significance. This value was appropriately adjusted by Bonferroni correction for taking into account the problems connected to multiple comparisons. The power analysis was performed in order to measure how the test is able to discriminate the groups. The statistical analysis was completed by assessing the power analysis on Z-test. Finally, the 95% confidence interval (CI) of the differences in the analyzed proportions was also calculated to evaluate the lower and upper bounds of the estimations.

## Results

**Figure 1:**
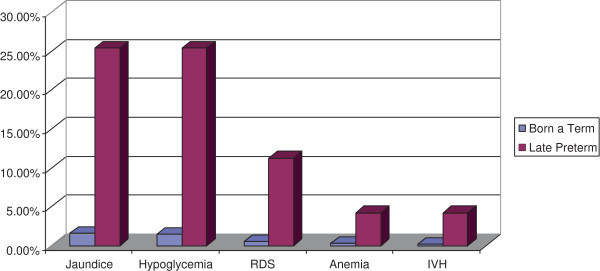
**Adverse neonatal outcomes in term and late preterm infants analyzed.** Data are expressed as percentages (%).

**Figure 2:**
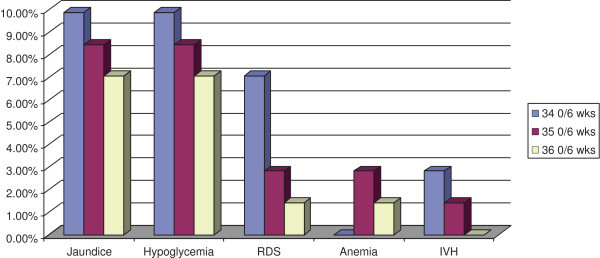
**Adverse neonatal outcomes in the late preterm infants stratified for gestational age.** Data are expressed as percentages (%).

1073 full-term deliveries and 71 late preterm deliveries occurred, in our division, during the sampling period. 185 infants (17.24%) among term deliveries, and 58 infants (81.69%) among late preterms were admitted to the NICU. Among full-term infants, 44 out of 1073 (4.10%) presented one major adverse outcome: 17 cases of jaundice (1.58%), 16 cases of hypoglycemia (1,49%), 6 cases of RDS (0.56%), 3 cases of anemia (0.28%) and 2 cases of intraventricular hemorrhage (0.19%) (Figure 1). Among late preterm infants, 49 out of 71 (69.01%) presented one major adverse outcome: 18 cases (25.35%) of jaundice, 18 cases (25.35%) of hypoglycemia, 8 cases (11.26%) of RDS , 3 cases (4.22%) of anemia and 3 cases (4.22%) of intraventricular hemorrhage (Figure 1).In the Figure 2 are represented adverse neonatal outcomes in the late preterm infants stratified for gestational age.

The Z-test analysis showed a significant increase in the incidence of hypoglycaemia and jaundice in all the 3 late preterms sub-groups compared to the term infants group (p < 0.0001). The incidence of RDS and of anemia was significantly increased in group A (p <0.0001; p = 0.008 respectively) and in group B (p = 0.003; p < 0.0001 respectively). Finally we found a statistically significant increase of IVH only in the sub-group A (p < 0.0001) (Table 1). A power of 70% was evaluated, indicating the ability of the test to highlight true differences among the analysed groups.

**Table 1:** **Statistical analysis of incidence of neonatal major complications in late preterms compared to born at term, in total and stratified for gestational age**

Pre-term vs term	34 ^0/6^ wks	35 ^0/6^ wks	36 ^6/7^ wks	Total
**Variable**				
Hypoglycemia	< 0.0001	< 0.0001	< 0.0001	< 0.0001
	9,86%	8,45%	7,04%	25,35%
Jaundice	< 0.0001	< 0.0001	< 0.0001	< 0.0001
	9,86%	8,45%	7,04%	25,35%
RDS	< 0.0001	0.003	0.630	< 0.0001
	7,04%	2,82%	1,4%	11,26
Anemia	0.008	< 0.0001	0.106	0.027
	0%	2,82%	1,41%	4,23%
IVH	< 0.0001	0.039	0.106	0.027
	2,82%	1,41%	0%	4,23%

A p value < 0,05 was considered statistical significant. In brackets the percentages (%).

## Discussion

The late preterm infant has historically been perceived to have similar risks for developmental problems as neonates born at term. However several studies, designed to observe clinical outcomes of late preterm births, have raised concern that these infants are at significantly increased risk, compared to born at term, of medical complications such as hypoglycemia, respiratory distress, anemia, jaundice and intraventricular hemorrhage (Committee on Obstetric Practice 2008; Wang et al. 2004; Escobar et al. 2006). In this study we analyzed the risk of adverse neonatal outcomes in late preterm infants born in our department, and we found, according to data published in literature (Consortium on Safe Labor et al. 2010; Kamath-Rayne et al. 2012), an increased incidence of morbidity when compared with those born at term (Figure 1). Moreover our data have been stratified for gestational age at birth, in order to better understand the contribution of each gestational age to neonatal morbidity and, interestingly we found that IVH occurred in 4,22% of total late preterms reaching statistical significance only at 34^0/6^ weeks of gestation. The incidence of anemia and RDS was significantly higher at 34 ^0/6^ and 35 ^0/6^ weeks of gestation when compared to full-term infants, on the contrary newborns of group C showed a risk of such complication, comparable to born at term. Finally, the incidence of hypoglycemia and jaundice results significantly higher in all the 3 sub groups of late preterms, compared to full term infants (Table 1). So we can suggest that the gestational age at delivery of late preterms can influence the risk of major complications. A recent multicentric study, conducted on a large cohort of deliveries in the United States (Consortium on Safe Labor et al. 2010) analyzed the short-term neonatal outcomes in late preterms, limited to the incidence of respiratory morbidity compared with term infants. In this study patients enrolled where stratified for gestational age and results demonstrated an increased risk of respiratory distress syndrome in all the subgroups of preterm’s gestational age. Our data are partially in agreement with the above mentioned study (Consortium on Safe Labor et al. 2010) (Table 1). In fact results showed an increased risk of RDS in late preterms of group A and B but we did not found a significant incidence of such complication in late preterms born at 36 weeks of gestation. The reason of this discrepancy is probably due to the exclusion from our study, of pregnancies complicated by diabetes, hypertension, and other obstetric complications as well as to the smaller number of cases analyzed.

Preterm birth has a significant financial impact too. In fact the total cost of late preterm births is derived from the cost of antepartum management, delivery costs, neonatal treatment costs, and the need for long term medical services for many of these infants (Loftin et al. 2010). On the basis of our finding reflecting current literature, obstetric care providers should reevaluate the need for delivery during the late preterm period (34 ^0/6^ to 36 ^0/6^ weeks) considering that the morbidity of the newborns at 34 and 35 weeks of gestational age is significantly different from newborns at 36 weeks. Instead, tocolytic agents, even for a short term treatment, could postpone preterm delivery long enough to improve neonatal outcome. In conclusion, results of our study demonstrated that morbidity in the late preterm period is clearly increased compared with those born at term, but in late preterm group the likelihood of significant neonatal morbidity decreases with advancing gestational age. Our findings underline the risk of IVH until 34 week of gestational age, and the risk of RDS until 35 weeks of gestation. Further investigations are needed to confirm these preliminary results to establish the role and the safety of tocolytic treatment in the different subgroups of late preterms infants.
